# Assessment of blood consumption score for pediatrics predicts transfusion requirements for children with trauma

**DOI:** 10.1097/MD.0000000000025014

**Published:** 2021-03-05

**Authors:** Akira Komori, Hiroki Iriyama, Makoto Aoki, Gautam A. Deshpande, Daizoh Saitoh, Toshio Naito, Toshikazu Abe

**Affiliations:** a Department of General Medicine, Juntendo University, Tokyo; b Department of Emergency Medicine, Gunma University Graduate School of Medicine, Maebashi; c Department of Traumatology and Emergency Medicine, National Defense Medical College, Tokorozawa; d Department of Emergency and Critical Care Medicine, Tsukuba Memorial Hospital; e Department of Health Services Research, Faculty of Medicine, University of Tsukuba, Tsukuba, Japan.

**Keywords:** blood transfusion, decision support techniques, focused assessment with sonography for trauma, Japan, vital signs, wound and injuries

## Abstract

Although transfusion is a primary life-saving technique, the assessment of transfusion requirements in children with trauma at an early stage is challenging. We aimed to develop a scoring system for predicting transfusion requirements in children with trauma.

This was a case–control study that employed a nationwide registry of patients with trauma (Japan Trauma Data Bank) and included patients aged <16 years with blunt trauma between 2004 and 2015. An assessment of blood consumption score for pediatrics (ped-ABC score) was developed based on previous literatures and clinical relevance. One point was assigned for each of the following criteria: systolic blood pressure ≤90 mm Hg, heart rate ≥120/min, Glasgow coma scale (GCS) score <15, and positive focused assessment with sonography for trauma (FAST) scan. For sensitivity analysis, we assessed age-adjusted ped-ABC scores using cutoff points for different ages.

Among 5943 pediatric patients with trauma, 540 patients had transfusion within 24 hours after trauma. The in-hospital mortality rate was 2.6% (145/5615). The transfusion rate increased from 7.6% (430/5631) to 35.3% (110/312) in patients with systolic blood pressure ≤90 mm Hg (1 point), from 6.1% (276/4504) to 18.3% (264/1439) in patients with heart rate ≥120/min (1 point), from 4.1% (130/3198) to 14.9% (410/2745) in patients with disturbance of consciousness with GCS score <15 (1 point), and from 7.4% (400/5380) to 24.9% (140/563) in patients with positive FAST scan (1 point). Ped-ABC scores of 0, 1, 2, 3, and 4 points were associated with transfusion rates of 2.2% (48/2210), 7.5% (198/2628), 19.8% (181/912), 53.3% (88/165), and 89.3% (25/28), respectively. After age adjustment, c-statistic was 0.76 (95% confidence interval, 0.74–0.78).

The ped-ABC score using vital signs and FAST scan may be helpful in predicting the requirement for transfusion within 24 hours in children with trauma.

## 1 Introduction

Trauma is a leading cause of death among young populations across the world.^[1,2]^ Even if most injuries of children are of mild-to-moderate severity,^[3]^ rapid evaluation and management of children with serious and life-threatening trauma are needed to avoid preventable trauma death.

Transfusion is one of the key life-saving elements for children with trauma. Delay in transfusion is primarily associated with increased mortality.^[4,5]^ However, it has been challenging for clinicians to assess the requirements for transfusion among children with trauma at an early stage.^[6]^ In addition, clinicians may hesitate to use transfusion for children owing to the risks of transfusion-related complications such as infection or allergic reaction.^[7]^

Several transfusion prediction scoring systems are available for patients with trauma.^[8,9]^ The majority of these systems were developed for adults and, subsequently, applied to pediatric populations; however, their effectiveness may be limited in children.^[10]^ Indeed, most transfusions for pediatric patients with trauma were decided without clear indications.^[11]^ There is no prediction scoring system focusing on blood transfusion in children with trauma.

Therefore, the objective of this study was to develop a scoring system to predict the requirements for transfusion in children with trauma.

## 2 Methods

### 2.1 Design and data collection

This was a case–control study that employed a nationwide registry of patients with trauma in Japan: Japan Trauma Data Bank (JTDB). JTDB was established in 2003 and is authorized and maintained by the Japanese Association for the Surgery of Trauma and the Japanese Association for Acute Medicine to improve and assure the quality of trauma care in Japan. A total of 272 hospitals, including >95% of certified tertiary emergency medical centers in Japan, contributed to JTDB in March 2018.^[12]^ JTDB records data regarding patient demographics, trauma causes, injury severity score (ISS), vital signs, and emergency procedures at pre-hospital on arrival, during the hospital stay, and treatment and emergency procedures including transfusion within 24 hours. It also records outcome data such as emergency department (ED) mortality, in-hospital mortality, and length of hospital stay.

### 2.2 Patient selection

**Figure 1:**
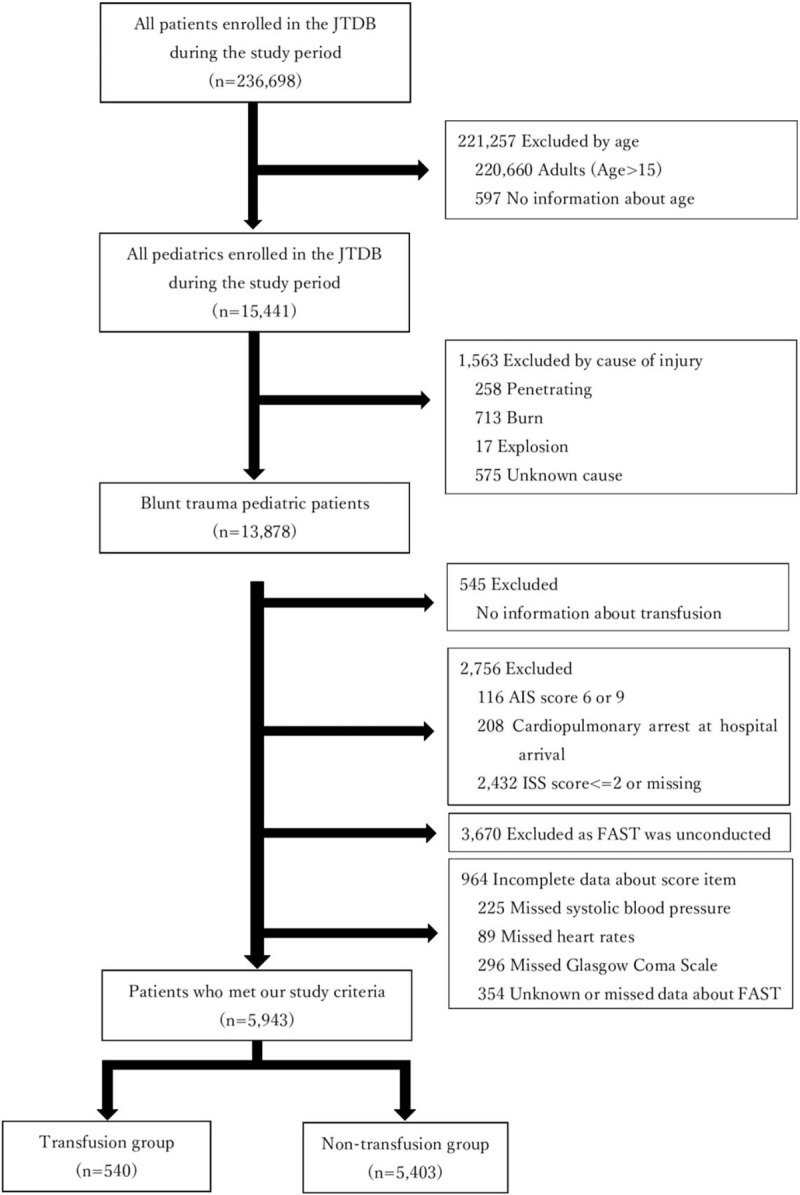
Flowchart of study participants.

All patients aged <16 years of age with blunt trauma were included (Fig. 1). The exclusion criteria were as follows: patients who had missing data of age; patients who had trauma mechanisms other than blunt trauma or patients with missing data of the trauma mechanism; patients who had no information about transfusion; patients who experienced cardiorespiratory arrest upon arrival at the hospital; or patients with an abbreviated injury scale (AIS) score of ≤2 or 6 (i.e., non-survivable injury) for any reason. Patients for whom focused assessment with sonography for trauma (FAST) scan was not conducted or data were missing; patients with missing data of systolic blood pressure (SBP), heart rate (HR), and Glasgow coma scale (GCS) scores in the ED were also excluded. Thus, the data of patients who represented complete datasets for score predictors of SBP, HR, GCS, and FAST were included in the analysis.

### 2.3 Development of the prediction score

The assessment of blood consumption score for pediatrics (ped-ABC score) was developed based on previous literatures and clinical relevance (Table 1). It comprises some components of the ABC score, which was developed to predict massive transfusion for adult patients with trauma,^[9]^ as well as disturbance of consciousness, which we defined as GCS score <15. We considered how the association between disturbance of consciousness and transfusion is explained through physiological rationale: severe blood loss leads to decreased cerebral blood flow and perfusion, resulting in disturbance of consciousness.^[13]^ Finally, 1 point was scored for each of the following criteria: SBP ≤ 90 mm Hg; HR ≥ 120/min; GCS score <15; and positive FAST scan.

**Table 1:** Assessment of blood consumption score for pediatrics.

Score item	Scoring
Systolic blood pressure	
≤90 mm Hg	1
Not applicable to above	0
Heart rate	
≥120/min	1
Not applicable to above	0
Consciousness	
<15 points on Glasgow coma scale	1
Normal	0
FAST	
Positive	1
Negative	0

### 2.4 Statistical analysis

Continuous variables were expressed as the median and interquartile ranges, which were compared using the Mann–Whitney *U* test, or as mean ± standard deviation, which were compared using the *t* test as appropriate. Categorical variables were expressed as numbers and percentages and compared using either the Chi-square test or Fisher exact test. Scoring items were assessed using c-statistic with 95% confidence interval (CI), and the characteristics were evaluated using sensitivity, specificity, and positive and negative predictive values for the score cutoff values of 1, 2, 3, and 4, respectively. For sensitivity analysis, we also assessed the age-adjusted ped-ABC score by using cutoff points for different age categories because the normal range of vital signs among pediatric populations differ depending on age.^[6,14]^ Normal vital signs and cutoff points were based on published normal ranges compiled from pediatric textbooks and guidelines.^[15,16]^ In the age-adjusted ped-ABC score, 1 point was scored for each of the following criteria: SBP ≤ 70 mm Hg plus child's age multiplied by 2 (age ≤10 years) or ≤90 mm Hg (age >10 years); HR ≥ 160/min (age ≤1 years), ≥150/min (1 < age ≤ 2 years), ≥140/min (3 ≤ age ≤ 5 years), ≥120/min (6 ≤ age ≤ 12 years), or ≥100/min (age ≥13 years); GCS <15; and positive FAST scan. We also conducted subgroup analyses for patients with ISS ≥15, patients without isolated head injury, and patients without severe isolated head injury (ISS ≥3 on head score of AIS). Patients with isolated head injury were considered to have received transfusion mainly for surgery.

For all analyses, a *P*-value of <.05 was considered statistically significant. All statistical analyses were performed using EZR (version 1.38; Saitama Medical Center, Jichi Medical University, Saitama, Japan), a graphical user interface for R (version 3.5.0; The R Foundation for Statistical Computing, Vienna, Austria).^[17]^ EZR is a modified version of R commander designed to apply statistical functions frequently used in biostatistics.

### 2.5 Ethics approval and consent to participate

We received permission to use the data from the steering committee of JTDB. The study protocol was reviewed and approved by the ethics committees of Juntendo University Hospital. The ethics committees waived the need to obtain informed consent from the study participants, given the retrospective and anonymized nature of this study. All methods were conducted in accordance with the relevant guidelines and regulations.

## 3 Results

### 3.1 Clinical characteristics

The JTDB enrolled 236,698 patients between January 2004 and December 2015. Of these, 5943 pediatric patients with trauma (2.5%) were eligible for assessment in our study (Table 2). In total, 540 patients (9.1%) received transfusion within 24 hours after trauma. ISS was higher in patients who received transfusion than in those who did not receive transfusion (25 [16–34] vs 10 [9–17], *P* < .01). Patients who received transfusion had higher HR (118 [94–142] vs 100 [85–116], *P* < .01), lower SBP (114 [98–132] vs 120 [110–132], *P* < .01), and lower GCS scores (10 [6–14] vs 15 [13–15], *P* < .01) than those who did not receive transfusion. A higher number of patients who received transfusion had positive FAST scan results compared with patients who did not receive transfusion (140 [25.9%] vs 423 [7.8%], *P* < .01). The overall in-hospital mortality rate was 2.6% (145/5615). The mortality rates of patients who received transfusion and those who did not receive transfusion were 20.2% (100/494) and 0.9% (45/5121), respectively.

**Table 2:** Characteristics of patients.

Characteristic	Transfusion (n = 540)	Non-transfusion (n = 5,403)	*P*-value
Age—yr	8.6 (±4.6)	9.2 (±4.0)	<.01
Male gender	364 (67.4)	3773 (69.8)	.31
Causes of trauma			<.01
Traffic accident	350 (64.8)	3588 (66.4)	
Sports	6 (1.1)	208 (3.8)	
Fall	156 (28.9)	1437 (26.6)	
Other blunt trauma	28 (5.2)	170 (3.1)	
AIS			
Head (n = 3627)	4 (3–5)	3 (2–4)	<.01
Face (n = 1482)	2 (1–2)	1 (1–2)	<.01
Neck (n = 41)	2 (1–3)	1 (1–1)	.13
Thorax (n = 1442)	4 (3–4)	3 (3–4)	<.01
Abdomen and pelvis (n = 1278)	3 (2–4)	2 (2–3)	<.01
Cervical spine (n = 376)	2 (2–3)	2 (2–3)	.20
Upper extremity (n = 1454)	2 (2–3)	2 (1–2)	<.01
Lower extremity (n = 1985)	3 (2–3)	2 (1–3)	<.01
Others (n = 407)	1 (1–1)	1 (1–1)	.31
ISS	25 (16–34)	10 (9–17)	<.01
Vital sign at ED			
HR, /min	118 (94–142)	100 (85–116)	<.01
SBP, mm Hg	114 (98–132)	120 (110–132)	<.01
RR, /min	26 (20–31)	24 (20–28)	<.01
GCS	10 (6–14)	15 (13–15)	<.01
Temperature, °C	36.4 (35.8–36.9)	36.7 (36.2–37.1)	<.01
FAST			<.01
Positive	140 (25.9)	423 (7.8)	
Negative	400 (74.1)	4980 (92.2)	
RTS	6.6 (5.4–7.6)	7.8 (7.6–7.8)	<.01
TRISS	0.94 (0.73–0.98)	0.99 (0.98–0.99)	<.01
Mortality (overall)	100 (20.2)	45 (0.9)	<.01
Mortality in ED or OR	7 (1.3)	7 (0.1)	
Discharge disposition			<.01
Home	244 (63.0)	4183 (82.8)	
Inter-hospital transfer	139 (35.9)	835 (16.5)	
Other	4 (1.0)	32 (0.6)	

Table 3 shows the distribution of patients by score items. The transfusion rate increased from 7.6% (430/5631) to 35.3% (110/312) in those with SBP ≤90 mm Hg (1 point); from 6.1% (276/4504) to 18.3% (264/1439) in those with HR ≥ 120/min (1 point); from 4.1% (130/3198) to 14.9% (410/2745) in those with disturbance of consciousness with GCS score <15 (1 point); and from 7.4% (400/5380) to 24.9% (140/563) in those with FAST positivity (1 point).

**Table 3:** Mortality and distribution of patients according to score item.

Score item	n (%)	Transfusion (%)	Mortality (%)
Systolic blood pressure			
≤90 mm Hg or <90	312 (5.2)	110 (35.3)	59/297 (19.9)
>90 mm Hg	5631 (94.8)	430 (7.6)	86/5318 (1.6)
Heart rate			
≥120/min	1439 (24.2)	264 (18.3)	70/1359 (5.2)
<120	4504 (75.8)	276 (6.1)	75/4256 (1.8)
Consciousness			
<15 points on GCS	2745 (46.2)	410 (14.9)	143/2588 (5.5)
Normal	3198 (53.8)	130 (4.1)	2/3027 (0.1)
FAST			
Positive	563 (9.5)	140 (24.9)	21/536 (3.9)
Negative	5380 (90.5)	400 (7.4)	124/5079 (2.4)

### 3.2 Predictive ability of ped-ABC score

The transfusion rates of patients based on their ped-ABC scores are presented in Table 4. Although patients with a score of 0 had a transfusion rate of only 2.2%, 89.3% of those with a maximum score of 4 received transfusion. Most patients scored <3 (96.7%).

**Table 4:** Transfusion of patients according to assessment of blood consumption score for pediatrics.

Score	n (%)	Transfusion by score, n (%)	Cumulative transfusion, n (%)
0	2210 (37.2)	48 (2.2)	48 (0.8)
1	2628 (44.2)	198 (7.5)	246 (4.1)
2	912 (15.3)	181 (19.8)	427 (7.2)
3	165 (2.8)	88 (53.3)	515 (8.7)
4	28 (0.5)	25 (89.3)	540 (9.1)

The score characteristics for transfusion rates in accordance with the different cutoff values are presented in Table 5. The specificity of patients with scores of 1, 2, 3, and 4 was 40.0%, 85.0%, 98.5%, and 99.9%, respectively. The sensitivity was 91.1% for patients with a score of 1, and the negative likelihood ratio for transfusion was 0.22 (95% CI, 0.17–0.29) and negative predictive value was 97.8% (95% CI, 97.2–98.3). For patients with a score of 3, the positive likelihood ratio for transfusion and positive predictive value were 14.1 (95% CI, 10.8–18.5) and 58.5% (95% CI, 51.9–64.9), respectively. In addition, for patients with a score of 4, the positive likelihood ratio for transfusion and positive predictive value were 83.4 (95% CI, 26.9–259.3) and 89.3% (95% CI, 72.9–96.3), respectively. The c-statistic of the score was 0.76 (95% CI, 0.74–0.78).

**Table 5:** Score characteristics for transfusion by varying score cut-offs.

Cut-off of score	Sensitivity (95% CI), %	Specificity (95% CI), %	PPV (95% CI), %	NPV (95% CI), %	LR+ (95% CI)	LR– (95% CI)
≥1	91.1 (88.5–93.2)	40.0 (40.0–40.0)	13.2 (12.8–13.5)	97.8 (97.2–98.3)	1.52 (1.47–1.56)	0.22 (0.17–0.29)
≥2	54.4 (50.6–58.2)	85.0 (84.6–85.4)	26.6 (24.7–28.5)	94.9 (94.5–95.3)	3.63 (3.29–3.98)	0.54 (0.49–0.58)
≥3	20.9 (18.5–23.2)	98.5 (98.3–98.7)	58.5 (51.9–64.9)	92.6 (92.4–92.8)	14.1 (10.8–18.5)	0.80 (0.78–0.83)
4	4.6 (3.8–5.0)	99.9 (99.9–1.00)	89.3 (72.9–96.3)	91.3 (91.2–91.3)	83.4 (26.9–259.3)	0.95 (0.95–0.96)

### 3.3 Sensitivity and subgroup analyses of ped-ABC scores

Supplemental Table 1, shows the number of patients and transfusion rate in each age category. After age adjustment using different cutoff points, c-statistic of the score was 0.76 (95% CI, 0.74–0.78), which was similar to that before age adjustment. The specificity of the score after age adjustment was higher than that before adjustment (Supplemental Tables 2 and 3, http://links.lww.com/MD/F830). The other analyses also showed similar test characteristics with respect to the original score (Supplemental Tables 4–9).

## 4 Discussion

### 4.1 Brief summary

We developed a scoring system to predict the requirements for transfusion in children with trauma by using a nationwide registry of patients with trauma in Japan. The results of SBP, HR, GCS score, and FAST scan were included in the scoring system. The scoring system will be helpful for clinicians to make a systematic evaluation.

### 4.2 Development of the ped-ABC score

We developed the ped-ABC score, which consists of vital signs and FAST scan results. Tachycardia and hypotension as well as low GCS scores have been associated with poor outcomes in pediatric patients with trauma.^[18–20]^ In addition, positive FAST scan results strongly suggest intra-abdominal injury, which is one of the main reasons for the requirement for transfusion.^[21,22]^ Scores with multiple combinations of vital sings have better predictability than vital signs alone.^[23]^

The use of indicators that utilize vital signs tends to be complicated in children because the normal range of vital signs varies with age. In this study, sensitivity analysis performed after age adjustment demonstrated that the test characteristics were equivalent to those before adjustment. Therefore, it is possible to adapt this scoring system more easily in children with trauma without setting different cutoff values for each age. In the subgroup analyses, we confirmed that the scores of patients with ISS ≥15, patients without isolated head injury, and patients without severe isolated head injury were equivalent to the original score. Establishing a scoring system also makes it easier for clinicians to assess the requirement for transfusions in a busy ED.

### 4.3 Comparison with previous studies

Previous studies have reported that shock index (SI), which is calculated as normal HR divided by SBP, predicted mortality in pediatric patients with trauma and served as an indicator of the requirement for transfusion.^[24,25]^ The prediction of the requirement for blood transfusion using SI may be used because of its high negative predictive value. The strength of our scoring system lies in the systematic evaluation of making a decision of transfusion based on the score. The ped-ABC score is composed of 4 points, whereas SI is composed of 2 choices: “yes” or “no.” It can be used not only for rule-out but also for rule-in. Moreover, our scoring system does not require cutoff points that vary according to the patient's age. In addition, our scoring system is noninvasive and can be used quickly, whereas other studies require laboratory tests.^[26,27]^ Further studies are needed to assess our scoring system.

### 4.4 Ability, utility, and implementation of ped-ABC score

The ped-ABC score may be better than the clinical gestalt.^[28]^ Clinicians are sometimes forced to choose between delaying transfusion and risking transfusion-related complications. Indeed, the majority of pediatric arrivals with trauma do not initially show clear indications for transfusion.^[11]^ Our scoring system enables clinicians to evaluate or discuss the need for transfusion using common criteria. Based on the ped-ABC score, blood transfusion might be reasonable for patients with a score of 3 or 4. However, it may be more debatable when a clinician examines patients with a score of 2, although some over-triage for transfusion might be tolerated from the clinical perspective, especially for children. Delaying transfusion until vital signs worsen or coagulopathy occurs in the course of examination would be an alternative to performing transfusion. Ultimately, a final decision regarding transfusion is needed. Although the scoring system may not have the ability to predict outcomes perfectly, it can still help clinicians to perform systematic evaluation through a simplified method.

### 4.5 Limitations

This study had several limitations. First, approximately 30% of data of FAST scan were not recorded. These might have been the cases in which a clinician considered the FAST scan unnecessary because of the mild state or because they shared a critical status. However, in these patients, the scoring system was not required to evaluate the requirement of blood transfusion. Second, a validation study is needed because this study was retrospective. Third, there might have been an indication bias of transfusion because we did not have the data of appropriateness of treatments. In 2002, a guideline for trauma care named Japan Advanced Trauma Evaluation and Care that was created with reference to the advanced trauma life support practice theory was introduced in Japan. In addition, all participating institutions were national-certified emergency centers. Therefore, we believe that most patients received appropriate treatments. Fourth, data about transfusion history was missing; however, as this is a very small proportion (3.9%), we consider it to have negligible effects on the results. Fifth, the requirement for transfusion is not the same as the urgency or the appropriateness of transfusion. Further studies are warranted to evaluate whether the ped-ABC score can reduce the time to implement transfusion and improve patient outcomes. Sixth, we focused on the simplicity of the scoring system. Consequently, the scoring system's c-statistic may not be very high. However, we believe that our score is useful as it enables fast and easy assessment at an early stage of trauma survey.

## 5 Conclusions

We developed the ped-ABC score: a scoring system to predict the requirement for transfusion with 24 hours in children with trauma using vital signs and FAST scan results.

## Acknowledgment

The authors thank Enago (https://www.enago.jp) for the final English language editing.

## Author contributions

**Conceptualization:** Akira Komori, Hiroki Iriyama, Toshikazu Abe.

**Data curation:** Akira Komori, Toshikazu Abe.

**Formal analysis:** Akira Komori.

**Methodology:** Akira Komori, Hiroki Iriyama, Makoto Aoki, Toshikazu Abe.

**Resources:** Daizoh Saitoh, Toshikazu Abe.

**Supervision:** Toshikazu Abe.

**Visualization:** Gautam A. Deshpande.

**Writing – original draft:** Akira Komori.

**Writing – review & editing:** Hiroki Iriyama, Makoto Aoki, Gautam A. Deshpande, Daizoh Saitoh, Toshio Naito, Toshikazu Abe.

## Supplementary Material

Supplemental Digital Content

## Supplementary Material

Supplemental Digital Content

## Supplementary Material

Supplemental Digital Content

## Supplementary Material

Supplemental Digital Content
