# Supplementary material for: Assessment of blood consumption score for pediatrics predicts transfusion requirements for children with trauma
Source: Medicine (Baltimore). 2021 Mar 5;100(9):e25014. doi: 10.1097/MD.0000000000025014 (PMC7939166; doi:10.1097/MD.0000000000025014)
Supplement: Supplemental Digital Content [file medi-100-e25014-s001.docx]

**Supplemental Table 1:** The number of patients and transfusion rate for each age category

| Age (years) | n (%) | Transfusion by each category, n (%) | Cumulative transfusion, n (%) |
| --- | --- | --- | --- |
| Infant  Age ≤ 1 | 233 (3.9) | 50 (21.5) | 50 (0.8) |
| Toddler  1 < Age ≤ 2 | 185 (3.1) | 32 (17.3) | 82 (1.4) |
| Preschool  3 ≤ Age ≤ 5 | 666 (11.2) | 54 (8.1) | 136 (2.3) |
| School Age  6 ≤ Age ≤ 12 | 3301 (55.5) | 246 (7.5) | 382 (6.4) |
| Adolescent  Age ≥ 13 | 1558 (26.2) | 158 (10.1) | 540 (9.1) |
