# Supplementary material for: Assessment of blood consumption score for pediatrics predicts transfusion requirements for children with trauma
Source: Medicine (Baltimore). 2021 Mar 5;100(9):e25014. doi: 10.1097/MD.0000000000025014 (PMC7939166; doi:10.1097/MD.0000000000025014)
Supplement: Supplemental Digital Content [file medi-100-e25014-s002.docx]

**Supplemental Table 2:** Transfusion of patients according to age-adjusted Assessment of Blood Consumption score for Pediatrics (n=5,943)

| Score | n (%) | Transfusion by score, n (%) | Cumulative transfusion, n (%) |
| --- | --- | --- | --- |
| 0 | 2,266 (38.1) | 41 (1.8) | 41 (0.7) |
| 1 | 2,610 (43.9) | 226 (8.7) | 267 (4.5) |
| 2 | 908 (15.3) | 171 (18.8) | 438 (7.4) |
| 3 | 141 (2.4) | 86 (61.0) | 524 (8.8) |
| 4 | 18 (0.3) | 16 (88.9) | 540 (9.1) |
