# Supplementary material for: Assessment of blood consumption score for pediatrics predicts transfusion requirements for children with trauma
Source: Medicine (Baltimore). 2021 Mar 5;100(9):e25014. doi: 10.1097/MD.0000000000025014 (PMC7939166; doi:10.1097/MD.0000000000025014)
Supplement: Supplemental Digital Content [file medi-100-e25014-s003.docx]

**Supplemental Table 3:** Score characteristics for transfusion according to different cut-offs for age-adjusted Assessment of Blood Consumption score for Pediatrics (n=5,943)

| Cut-off of score | Sensitivity  (95% CI), % | Specificity  (95% CI), % | PPV  (95% CI), % | NPV  (95% CI), % | LR+  (95% CI) | LR-  (95% CI) |
| --- | --- | --- | --- | --- | --- | --- |
| ≥1 | 92.4 (88.9–94.3) | 41.2 (40.9–41.4) | 13.6 (13.2–13.9) | 98.2 (97.6–98.7) | 1.57 (1.52–1.61) | 0.18 (0.14–0.25) |
| ≥2 | 50.6 (46.7–54.4) | 85.3 (84.9–85.7) | 25.6 (23.6–27.5) | 94.5 (94.1–94.9) | 3.44 (3.10–3.80) | 0.58 (0.53–0.63) |
| ≥3 | 18.9 (16.7–20.9) | 98.9 (98.7–99.1) | 64.2 (56.8–70.9) | 92.4 (92.2–92.6) | 17.9 (13.2–24.4) | 0.82 (0.80–0.84) |
| 4 | 3.0 (2.2–3.2) | 1.00 (99.9–1.00) | 88.9 (67.3–96.9) | 91.2 (91.1–91.2) | 80.0 (20.6–312.3) | 0.97 (0.97–0.98) |

C-statistic of the score: 0.76 (95%CI, 0.74–0.78)
