# Supplementary material for: Assessment of blood consumption score for pediatrics predicts transfusion requirements for children with trauma
Source: Medicine (Baltimore). 2021 Mar 5;100(9):e25014. doi: 10.1097/MD.0000000000025014 (PMC7939166; doi:10.1097/MD.0000000000025014)
Supplement: Supplemental Digital Content [file medi-100-e25014-s004.docx]

**Supplemental Table 4:** Transfusion of patients who had Injury Severity Score ≥ 15 according to Assessment of Blood Consumption score for Pediatrics (n=2,540)

| Score | n (%) | Transfusion by score, n (%) | Cumulative transfusion, n (%) |
| --- | --- | --- | --- |
| 0 | 482 (19.0) | 20 (4.1) | 20 (0.8) |
| 1 | 1,304 (51.3) | 160 (12.3) | 180 (7.1) |
| 2 | 594 (23.4) | 159 (26.8) | 339 (13.3) |
| 3 | 134 (5.3) | 78 (58.2) | 417 (16.4) |
| 4 | 26 (1.0) | 23 (88.5) | 440 (17.3) |

**Supplemental Table 5:** Score characteristics for transfusion according to different cut-offs for Assessment of Blood Consumption score for Pediatrics in patients who had Injury Severity Score ≥ 15 (n=2,540)

| Cut-off of score | Sensitivity, % | Specificity, % |
| --- | --- | --- |
| ≥1 | 95.5 | 22.0 |
| ≥2 | 59.1 | 76.5 |
| ≥3 | 23.0 | 97.2 |
| 4 | 5.2 | 99.9 |

C-statistic of the score: 0.72. 95% CI: 0.70–0.75

**Supplemental Table 6:** Transfusion of patients without isolated head injury according to Assessment of Blood Consumption score for Pediatrics (n=4,715)

| Score | n (%) | Transfusion by score, n (%) | Cumulative transfusion, n (%) |
| --- | --- | --- | --- |
| 0 | 1867 (39.6) | 47 (2.5) | 47 (1.0) |
| 1 | 1967 (41.7) | 152 (7.7) | 199 (4.2) |
| 2 | 707 (15.0) | 153 (21.6) | 352 (7.5) |
| 3 | 146 (3.1) | 80 (54.8) | 432 (9.2) |
| 4 | 28 (0.6) | 25 (89.3) | 457 (9.7) |

**Supplemental Table 7:** Score characteristics for transfusion according to different cut-offs for Assessment of Blood Consumption score for Pediatrics in patients without isolated head injury (n=4,715)

| Cut-off of score | Sensitivity, % | Specificity, % |
| --- | --- | --- |
| ≥1 | 89.7 | 42.7 |
| ≥2 | 56.5 | 85.4 |
| ≥3 | 23.0 | 98.4 |
| 4 | 5.5 | 99.9 |

C-statistic of the score: 0.77. 95% CI: 0.75–0.79

**Supplemental Table 8:** Transfusion of patients without severe isolated head injury according to Assessment of Blood Consumption score for Pediatrics (n=4,923)

| Score | n (%) | Transfusion by score, n (%) | Cumulative transfusion, n (%) |
| --- | --- | --- | --- |
| 0 | 1,972 (40.1) | 47 (2.4) | 47 (1.0) |
| 1 | 2,045 (41.5) | 152 (7.4) | 199 (4.0) |
| 2 | 732 (14.9) | 153 (20.9) | 352 (7.2) |
| 3 | 146 (3.0) | 80 (54.8) | 432 (8.8) |
| 4 | 28 (0.6) | 25 (89.3) | 457 (9.3) |

**Supplemental Table 9:** Score characteristics for transfusion according to different cut-offs for Assessment of Blood Consumption score for Pediatrics in patients without severe isolated head injury (n=4,923)

| Cut-off score | Sensitivity, % | Specificity, % |
| --- | --- | --- |
| ≥1 | 89.7 | 43.1 |
| ≥2 | 56.5 | 85.5 |
| ≥3 | 23.0 | 98.5 |
| 4 | 5.5 | 99.9 |

C-statistic of the score: 0.77. 95% CI: 0.75–0.80
